# Can a structured model of ethical reflection be used to teach ethics to nursing students? An approach to teaching nursing students a tool for systematic ethical reflection

**DOI:** 10.1002/nop2.1339

**Published:** 2022-09-12

**Authors:** Lena Marian Jakobsen, Kjersti Sunde Mæhre

**Affiliations:** ^1^ Department of Health and Care Sciences, Faculty of Health Sciences UiT The Arctic University of Norway Tromso Norway

**Keywords:** ethical reflection, ethics in nursing programmes, nursing students, reflection groups, structured ethical reflection model

## Abstract

**Background:**

Nurses encounter many ethical dilemmas in their practice. The ability to make good ethical decisions is a necessary competence in healthcare professions. International studies call for development and research on various methods to teach healthcare professionals ethics. This article describes an approach for learning how to be aware of and discover ethical dilemmas. By applying experienced narratives from healthcare practice and using question guidelines from a structured ethical model, nursing students learn to discover and find possible solutions for ethical problem in their practice.

**Aim:**

The aim of this study was to describe second‐ and third‐year nursing students’ experiences by using structured ethical reflection as an approach to increase ethical awareness and deal with ethical decisions.

**Design:**

This study has a descriptive exploratory design. A three‐day ethics seminar was carried out to help students learn how to recognize and explore ethical dilemmas in their practice.

**Materials and Methods:**

The data are collected from questionnaires used to evaluate the ethics seminar where 52 nursing students participated. The questionnaire contained open‐ended and closed questions and was analysed using Braun and Clarke’s reflexive thematic analysis.

The empirical data were collected by 52 nursing students answering an evaluation questionnaire after the ethics seminar.

**Findings:**

Four themes were developed: *Becoming aware of ethical dilemmas, Learning ethics by discussing knowledge and experiences with other students, Increased curiosity about ethics as a subject* and *Understanding the importance of critical ethical reflection work in clinical practice*.

**Discussion:**

The process of learning how to understand the ethical principles in real‐life nursing context continues progressing through the bachelor’s program. Using group discussions and discussing examples of ethical dilemmas from practice help the students to a more comprehensive reflection process.

**Conclusion:**

The nursing students experienced video lessons, group discussions and the use of a structured reflection model as a valuable approach in learning to recognize ethical dilemmas and how to deal with real‐life ethical dilemmas.

## 1 INTRODUCTION

Nurses need the ability to translate ethical principles into practical situations. However, ethics is often perceived as a difficult subject (Sari et al., 2018) and somewhat irrelevant, both by students (Løviknes & Struksnes, 2013) and by newly qualified nurses (Alvsvåg & Førland, 2006). One of the goals of the Framework Plan for Nursing Education (Norwegian Ministry of Education and Research) is as follows: “Students must be able to reflect on ethical issues, be prepared to act ethically and gain insight into value conflicts”. To develop teaching methods that can make the subject of ethics more interesting and comprehensible in relation to clinical practice, with a minimum of difficult theory, is an important task for educational institutions (Alvsvåg & Førland, 2006). Studies show that newly qualified nurses' encounter with the healthcare system can be demanding when their values and expectations are challenged (Hazelwood et al., 2017; Martins et al., 2021).

Ethical reflection is necessary for nursing students in order to prepare them for caring for vulnerable human beings. Having the ethical awareness necessary, to recognize ethical challenges and handle them is an important part of the curriculum. This study aims to enhance nursing students’ ethical capability by teach them to use a structural ethical reflection model.

## 2 BACKGROUND

White Paper No. 16 (2016–2017), Culture for Quality in Higher Education, states: “In‐depth and transformative learning must be provided where students, by gaining new perspectives, develop a qualitatively new understanding of phenomena and interrelationships, and knowledge‐based and critical evaluative judgment” (Norwegian Ministry of Education and Research, 2017). In transformative learning, reflection is an important part of the learning process (Spekkink & Jacobs, 2021), both in Norway and internationally (Shrivastava & Shrivastava, 2021). To find the motivation to change oneself, one’s values and preconceptions must be challenged. Reflection can help to change one’s perception of the world. In order to enhance one’s understanding of experiences gained from different situations in practice, reflecting on what has been experienced will provide a new understanding of the context and the whole picture (Dewey, 2008).

The development of good judgement (by Aristotle called phronesis) implies that a practitioner has both episteme (theoretical knowledge) and techne (practical skills) (Shorey, 1934). The practitioner needs phronesis in situations where she or he has to choose between actions, and where there are no clear answers as to what will be good decisions (Aristotele, 1999/2006). Reflection on one’s own experiences, actions and values can lead to new insights and new practices in professional studies (Hatlevik, 2018; Norwegian Ministry of Education and Research, 2019). Educational institutions must help students learn to critically reflect on their own practice, and thus become aware of the choices of action they take (Albert et al., 2020; Femdal et al., 2017; Mæland et al., 2021), and this must form part of programmes for nursing students’ (Norwegian Ministry of Education and Research, 2019). Heath (Heath, 1998) describes how ethical knowledge enhances reflection and helps nurses to make better choices in their practice. Teaching students to use an ethical reflection model will help them to recognize an ethical problem, and increase their understanding of how their own values and attitudes affect the actions they choose inpatient encounters.

A profession is characterized by how professional knowledge is expressed through reflection and action (Dahl & Alvsvåg, 2013). Nursing has its own ethical guidelines for professional practice; nurses thus have a professional, ethical and personal responsibility for their actions and assessments. However, students may need help to see and understand how nursing norms, rules and relevant legislation should be handled in clinical practice (Sari et al., 2018). The use of value‐based terminology and a common understanding of ethical concepts will be necessary to help students to formulate and clarify ethical values (Bie, 2020). Such an approach increases awareness of the importance of ethics in patient care (Nilsen et al., 2015). An evaluation conducted by the national project “Collaboration to enhance knowledge of ethics” in 2015 called for a greater focus on ethical reflection by educational institutions. This will benefit future nursing practice, where nurses are also expected to cooperate with colleagues on work involving ethical issues (Nilsen et al., 2015).

### 2.1 The structured reflection model of the Centre for Medical Ethics at the University of Oslo

In 2007–2015, the national project “Collaboration to Enhance Knowledge of Ethics in Primary Healthcare” was implemented in 243 local authorities in Norway. The goal was to strengthen ethics knowledge and skills in the healthcare sector (Karlsen, Gjerberg, et al., 2018). The Centre for Medical Ethics at the University of Oslo has designed a model for systematic ethical reflection known as the Centre for Medical Ethics (CMEs) model, which is used to teach primary healthcare personnel how to deal with ethical dilemmas (Magelssen et al., 2016). The Norwegian Association of Local and Regional Authorities (KS) has also drawn up a similar, six‐step reflection model in addition to instructions on how to understand the model (KS, the Norwegian Association of Local and Regional Authorities, 2021). Research on experience with the CME model in primary healthcare shows that it is well‐suited to practice (Karlsen, Gjerberg, et al., 2018; Karlsen, Lillemoen, et al., 2018).

The CME model is a dialogue‐based and pragmatic methodological approach to ethical reflection based on a moral dilemma or ethical issue taken from a case in practice (Paulsen et al., 2011). In the reflective dialogue, participants are encouraged to put their experiences into words. The aim is to challenge one’s own prejudices and to jointly explore different ethical views and values. Such joint exploration of the moral dilemma or issue from the case is intended to lead to one or more suggestions as to what may be the best course of action as well as a possible common moral understanding (Metselaar et al., 2015).

The research question is as follows: How can the CME model support the second‐ and third‐ year nursing students' ability to reflect and recognize and handle ethical challenges?

## 3 METHODS

This study had a qualitative exploratory descriptive design. Such an approach to research enables the researcher to gain insight into what the participants believe works or does not work in a realistic context (Hunter et al., 2019), and allows for the exploration and description of a phenomenon that has been studied little. This design made it possible to ascertain whether the ethics seminar with its focus on a structured reflection model was useful for nursing students learning ethics.

### 3.1 Data collection

The data for this article came from an online questionnaire the students were asked to complete after a 3 days ethics seminar as part of their curriculum. The students were encouraged to evaluate all the different parts of their study programmes. It was voluntary and anonymous to give feedback. The questions were both open‐ended and closed. The open‐ended questions were: (1) What have you learned during the seminar? (2) Do you feel that you can lead ethical reflection groups? (3) Have you any changes to suggest for the implementation of the seminar? (4) What do you want/need to learn more about? There were two closed questions: Do you feel that you can lead a reflection group after this seminar? and Do you feel that you have gained important knowledge about the use of ethical theory during this seminar? Both questions had three response options, such as “yes”, “a lot” and “some”. Two nursing school classes participated in this study, one in 2018 and the other in 2021. Due to the confidentiality regulations, we have not gathered specific demographic information.

### 3.2 Analysis

Variation in the empirical data (a few differences between questionnaires) necessitated a more flexible analysis method inspired by Braun and Clarke (2006); the method used was thus based on reflexive thematic analysis (Braun & Clarke, 2020). The two authors analyzed the data separately and then together. We first collected the answers to the closed questions, followed by an analysis of the responses to the open‐ended questions, where we elicited the essence of the students' feedback. Meaning units were then categorized and a close interpretation of the text led to sub‐themes. Next, we re‐read the themes, text, codes and sub‐themes, moving back and forth between them. Finally, we grouped sub‐themes into four themes: Becoming aware of ethical dilemmas, Learning ethics by discussing knowledge and experiences with other students, Increased curiosity about ethics as a subject and Understanding the importance of critical ethical reflection work in clinical practice.

### 3.3 Participants

Fifty two students (19 in their third year and 33 in their second year) attended the ethics seminar and all completed questionnaires after the seminar in 2018 and in 2021. The students were both women and men, but mainly women, aged 28 years on average.

### 3.4 Ethical statement

Students answered the questionnaires on a voluntary basis. The responses were anonymous and not personally identifiable, and the study did not require approval from the Norwegian Centre for Research Data. It is part of the nursing students’ programmes to evaluate their nursing programmes. The university recommends the teachers to use feedback from students’ evaluations in order to enhance teaching methods and content. The data were collected through “Nettskjema”—an encrypted dataservice programme at the University of Oslo. During the transcription, the questionnaires were anonymized with letter codes for “second‐year student” or “third‐year student”, which only the researchers/authors could recognize.

### 3.5 The three‐day ethics seminar

The seminar consisted of preparation, implementation and conclusion. Before it began, the students were asked to read ethical theory; various books and online resources were made available on the learning platform. The students were in their usual groups of 5–8 students each. Attendance at the seminar was compulsory.

Day 1 (implementation). Principles and concepts related to ethics and reflection were presented, and four short YouTube videos on various ethical theories were shown (duty ethics, the four principles of ethics, consequential ethics and utilitarian ethics). During the first 2 days, each group was given two different tasks: (a) To practise recognizing an ethical dilemma. and (b) To practise formulating an ethical issue. Students were given a total of 11 cases to work on, two in each group. They had 45 min to work on their cases before they were discussed in the whole class.

Day 2 began with a video from CME showing an example of an ethical dilemma in practice. The reflection model and the group leader’s key function were presented before the groups were each given a case based on those dealt with in a clinical ethics committee, from both secondary and primary healthcare. The students spent half of the day discussing their case using the six steps of the CME model (Table 1). Each student in a group was encouraged to be responsible for leading one of the steps in the model. In this way, all the students gained experience of leading an ethical reflection group. Two teachers provided advice and guidance to the groups.

**TABLE 1:** Example of a structured reflection model

Step 1	Step 2	Step 3	Step 4	Step 5	Step 6
Formulate the problem: What is the ethical dilemma/problem?	What are the relevant facts in the case?	Who are the parties involved in the case? What is their view of the case?	Which ethical principles and values are involved? What do laws and regulations say?	What are the possible choices? How can they be justified?	Which solution is best overall? What can the parties agree on?

Day 3 (conclusion): The groups presented to the whole class how they had chosen to solve the ethical dilemma in the case. After each presentation, there was an opportunity for discussion and input from other students. Finally, students were encouraged to answer the online questionnaire with its closed‐ and open‐ended questions.

## 4 RESULTS

First, we present and summarize the results from the closed questions. We then present the results from the open, descriptive responses where we used a semantic thematic analysis.

### 4.1 Summary of answers to closed questions

The following questions were asked: Do you feel that you can lead a reflection group after this seminar? (Table 2), and Do you feel that you have gained important knowledge about the use of ethical theory during this seminar? (Table 3).

**TABLE 2:** Students leading reflection groups

Do you feel that you can lead a reflection group after this seminar?	Yes	Maybe	No
Third‐year students	14	5	0
Second‐year students	17	8	8
All students	31	13	8

**TABLE 3:** Student’s knowledge of ethical theory

Do you feel that you have gained important knowledge about the use of ethical theory during this seminar?	Yes	A lot	Some
Third‐year students	17	0	2
Second‐year students	16	16	1
All students	33	16	3

Thus, many third‐year students reported having gained important knowledge of ethical theory during the seminar, but the learning outcome seems to be lower among second‐year students. There were also significantly more third‐year students who felt that the CME model had given them the skills to lead an ethical reflection group.

### 4.2 Open‐ended responses

The analysis of the open‐ended responses revealed four themes:

Becoming aware of ethical dilemmas, Learning ethics by discussing knowledge and experiences with other students, Increased curiosity about ethics as a subject and Understanding the importance of critical ethical reflection work in clinical practice.

#### 4.2.1 Becoming aware of ethical dilemmas

Mutual discussion of cases where students learned to recognize ethical dilemmas led to increased knowledge and awareness of how ethical issues can be solved. One third‐year student said: “I want more cases … I’ve become more aware that ethical decisions are challenging … A good procedure/model that leads to useful discussions and answers”. Another third‐year student said: “You learn a lot from hearing how the others have solved the cases, so you can have a good discussion in the whole class”. Most students understood what an ethical dilemma is and can be, but seeing it in the light of ethical theory was difficult for many of them, including third‐year students.

#### 4.2.2 Learning ethics by discussing knowledge and experiences with other students

Learning from others' experiences was felt to be very important, and many students said that this increased their knowledge and awareness of ethical issues. Some students, mainly from the third year, pointed out the benefit of being in reflection groups with other nursing students, but also felt that students from other professional programmes such as social work could make important contributions. They believed that such students could highlight other perspectives and suggest different solutions from those they had thought about themselves. Several students, mainly from the second year, initially stated that they did not want to be forced into group work; however, in the open‐ended responses most students answered that working in groups with the CME model and cases had actually taught them the most. Some students, almost entirely second‐year students, were sceptical about having to present their views on a case, but the responses to the questionnaire suggest that sharing experiences was still considered useful. A second‐year student wrote: “I felt that we learned from the group work, but the presentations seemed unnecessary. I didn’t learn any more from it, and you don’t discuss much in large groups”. However, there were students in both years who disagreed that they did not learn anything from the presentations to the whole class. A second‐year student wrote: “Listening to a presentation of the same topic by eight groups was a drag, but still it was useful to hear what perspectives the different groups used to find the best possible solution to the case”.

#### 4.2.3 Increased curiosity about ethics as a subject

Working on the cases, all previously presented in the clinical ethics committee, gave the students insight into the kinds of ethical issues that may arise in clinical practice and many students in both years wanted access to cases from the entire health and social care sector. One third‐year student said: “I’d like more variety of cases and more breadth in the ethical problems so that we can practise more practical ethical reflection. For example, more cases from various healthcare services such as nursing homes, home nursing, emergency services, hospital wards and doctors” surgeries’. Many students stated that they had good knowledge of ethics, but that it was difficult to translate ethical theory into practice to make well‐founded decisions. One second‐year student put it this way: “I want to learn more about ethical reflection and reflection in practice ‐ ethical theory that can easily be applied to practice”.

#### 4.2.4 Understanding the importance of critical ethical reflection work in clinical practice

Several third‐year students stated that the introduction and use of the CME model could have been provided earlier in their programme, preferably after the first‐year practice period. However, second‐year students were more divided in their responses as to when the learning outcome of the model would be greatest. Most students in both the years stated that the discussions with other students in groups were useful and led to learning. One third‐year student said: “It taught me a lot to look at the problem from several angles and to learn from each other”. Listening to each other’s opinions and experiences and suggesting solutions gave several students a new dimension to their reflection. Many said that the consequences of previously suggested choices of action had to be reconsidered, and perhaps changed if they could not be professionally justified. A second‐year student said: “The discussion brought out different views and possible consequences, which encouraged me to think and reflect more”. The students felt that the structured model challenged them to use the knowledge and skills they had learned as nursing students. A third‐year student said: “You get to use the knowledge you have learned from all the three years of our nursing programme”. Having the structured reflection model as their “guide” was important for many students. One third‐year student said: “The structured application of ethics has helped me to understand the subject better, and I see how we use this all the time without actually being aware of it”.

## 5 DISCUSSION

The aim of this study was to explore nursing students' experiences of an ethics seminar focusing on teaching ethical reflection with the help of a structured ethical reflection model (the CME model).

### 5.1 Becoming better equipped to deal with the complexity of everyday nursing

A practical approach to ethical reflection in the form of a structured model helped students to focus on the ethical problem and related factors that formed part of a case. Students’ feedback shows that it is easier to discuss ethical issues with the help of a template (Karlsen, Lillemoen, et al., 2018), which for our students meant answering the six questions in the structured reflection model (Table 1).

A systematic reflection model that starts with lived experience of problems taken from practice and moves on to an understanding of the problems based on ethical theory has, in previous studies, been shown to increase the ethical awareness and competence of healthcare workers (Rasoal et al., 2017), which we also found in our students. However, using a structured method of ethical reflection does not necessarily increase students' level of reflection. If students are to benefit from such an ethical reflection model, we found as in other studies that the issues must be relevant to practice and that students should have experience of similar situations (Dahl & Eriksen, 2016). During their practice placements, many of our students experienced ethically difficult situations and dilemmas (Heggestad et al., 2020), which made it easier to share examples and experiences with other students and teachers during the seminar.

Previous studies have shown that healthcare workers who have participated in systematic ethical reflection groups find it easier to deal with ethical challenges and have better collaboration with colleagues, patients and relatives (Antonsen et al., 2018). The nursing students found that the seminar provided them with ethical theory that could be put into practice. A majority believed that greater knowledge of ethics made them better equipped to lead ethical reflection groups, which would also be useful for them as qualified nurses. Studies have shown that leaders of ethical reflection groups should have knowledge and experience in leading ethics work in order to succeed with such groups (Antonsen et al., 2018; Widdershoven et al., 2016).

Students need to practise before they can understand and apply ethical principles in everyday work (Kim & Park, 2019), and this is best done through experience sharing. Practical reality is often more complex than we can understand based on the theoretical concepts alone (Molewijk et al., 2008), and the students' desire to work with a variety of cases showed that they had an understanding of the complexity. Learning in a social community made the students more motivated and engaged in their learning. Analytical reflection means challenging what we assume we understand. Steen Wackerhausen divides reflection work into two parts. First order reflection is problem solving, where we think within the concepts and discourses used in our profession. In the second order reflection, we are able to question the concepts and perspectives that we hold and act upon (Wackerhausen, 2008). Østerberg (1966) writes that an analysis “involves examining a whole by determining the parts with which it is composed as well as the relations between the parts” (p. 10). Reflection helps us to see the whole complex picture.

This will require input from other professions, because one may be blind to the idea of questioning usual, everyday phenomena within one’s own profession. We did not plan such input as part of our ethics seminar, but we see it as valuable and would like to try it out. Working with first and second order reflection as we have done meant that students were able to question their own professional practice. In the groups and the whole class, the students challenged each other by thinking critically about their practice (Vygotsky, 2001). Our study shows that third‐year students are better than second‐year students at applying ethical reflection to a clinical context. This may be because they have progressed further in their studies, with more theory and more practice periods, but it may also be because of a slightly exaggerated belief in their own moral competence (Koskinen et al., 2021) Reflections on the cases have provided all the students with a new understanding of the interrelationship between practice and theory (Dewey, 2008).

Reflection is necessary for learning. Reflecting with others implies not being afraid to challenge our own opinions and understandings. By putting together many small pieces, students will be able to see a greater connection between their own and others' values and attitudes, and thus develop everyday ethical skills and sound judgement (Bie, 2020).

Working in groups and discussing ethical issues was demanding for some students, mostly for those in their second year. Third‐year students asked questions more readily in the ethics seminar, and more often suggested possible actions to the other group members (Bie, 2020). Students need to feel safe in their group to share experience and knowledge and reflect with others (Bie, 2020; Dahl & Alvsvåg, 2013; Tutticci et al., 2020), and it has been shown that fewer students in each group improves learning (Pagnucci et al., 2015). When the group members felt more at ease with each other, both we and the students found that more mutual learning took place (Dewey, 2008). They were not afraid to express their understanding of the case, perhaps because they felt secure and received support from teachers and other students to understand how different ethical concepts, theories and legislation can be applied in a given situation and to make sound decisions on what action to take. The result was that even those who shared their experiences discovered things they had not understood previously. In a safe learning community, students find support to understand complexity (Dahl & Eriksen, 2016) in moral issues (Cannaerts et al., 2014). We found that more openness and greater focus on understanding a situation from different perspectives led to more sensible suggestions for action.

### 5.2 Moral awareness occurs through maturation

Florence Nightingale, the mother of nursing, argued that the patient’s own experiences should form the basis for sound decisions in nursing (Nightingale, 2003). Trying to understand the other, and being available to meet the other’s needs, requires practice in listening without judging or criticizing. It can certainly be difficult to listen and try to understand the other as different from oneself, but with imagination this can be practised both in an educational setting and in nursing (Martinsen, 2021). Several nursing students found it difficult to address ethical issues if they felt that something “didn’t seem right” in practice. In such uncertainty, students are afraid to speak out if they find that clinical practice is not in the patient’s best interests (Bickhoff et al., 2016). We found that the students gained confidence in offering their opinions and discussing attitudes as the seminar progressed. In particular, we noticed that recognizing ethical challenges was easier after they had attempted to put into words what was/was not an ethical issue. Moral courage implies that nursing students dare to speak up if they find that patient care is unsatisfactory (Gibson et al., 2020), and here too we noticed a positive development in the course of the seminar.

In their clinical practice, nurses will encounter ethical issues that are difficult to solve. Correct action in one situation may be detrimental in another situation even if the situations appear similar in many ways. Choosing the right action can be difficult, especially if none of the actions are optimal. For example, it can be challenging to make good choices for patients who lack autonomy; here, nurses have to collaborate with doctors and the patients' relatives on decision making on behalf of the patient (Edwards, 2009). As teachers, we found that third‐year students worked more independently with the structured reflection model, while second‐year students expressed more frustration at not understanding what we expected them to do. Many students, especially those in their third year, wished that the ethics seminar could have been arranged after the first practice period in their first year, because they now realized how ethical issues could have been handled in clinical practice. However, this study, even though the sample is limited, shows that second‐year nursing students do not have the academic and practical background to achieve the same learning outcomes as those in the third year (Manninen et al., 2020).

## 6 CONCLUSION

By combining different teaching methodologies and using the CME model as our basis in the ethics seminar, we found a sound method to teach students a structured approach to systematic ethical reflection. The model helped the students to understand the complexity of everyday clinical practice, to recognize an ethical issue and to choose a suitable professional course of action.

### 6.1 Strengths and limitations

It has been important for the researchers to provide a clear description of the analysis, since the questionnaires differed slightly. The use of a questionnaire may provide less feedback from students' experiences, but it still fulfilled the study aim. A larger number of ethics seminars and students might have further enhanced the data. However, this might have affected the transfer value to other teaching of students.

### 6.2 Relevance for nursing education and practice

In our view, learning a structured method of ethical reflection will help to make participants (as nursing students and later as nurses) aware of the value of improving ethics work in clinical practice. Cooperation between healthcare workers and the ability to listen and choose between alternative courses of action on ethical issues are important qualities, which can be learned through the use of a structured ethical reflection model.

## AUTHOR CONTRIBUTIONS

Both the authors have equally contributed throughout the process of writing this article.

All authors have agreed on the final version and meet at least one of the following criteria [recommended bythe ICMJE (http://www.icmje.org/recommendations/)]:

• substantial contributions to conception and design, acquisition of data or analysis and interpretation of data;

• drafting the article or revising it critically for important intellectual content.

## FUNDING INFORMATION

The study was conducted during time allotted to research and development at UiT The Arctic University of Norway. There was no external funding.

## CONFLICTS OF INTEREST

There are no conflicts of interest.

## Data Availability

The data that support the findings of this study are available from the corresponding author upon reasonable request.

## References

[nop21339-bib-0001] Albert, J. S. , Younas, A. , & Sana, S. (2020). Nursing students' ethical dilemmas regarding patient care: An integrative review. Nurse Education Today, 88, 104389. 10.1016/j.nedt.2020.104389 32193068

[nop21339-bib-0002] Alvsvåg, H. , & Førland, O. (2006). Sykepleierutdanningen i lys av nyutdannedes yrkeserfaringer [Nursing education in light of recent graduates' experiences from the workplace]. Vård i Norden, 26(3), 34–38.

[nop21339-bib-0003] Antonsen, Y. , Normann, A. K. , Nilsen, H. R. , & Magelssen, M. (2018). Systematisk etikkarbeid krever lederforankring [Systematic ethics work needs strong management involvement]. Tidsskrift for omsorgsforskning, 4(1), 40–49.

[nop21339-bib-0004] Aristotele . (1999/2006). Ethics. A major wor of Aristotle\s philosophy, also called “the Nicomachean” (Vol. 3). Gyldendal Akademisk.

[nop21339-bib-0005] Bickhoff, L. , Levett‐Jones, T. , & Sinclair, P. M. (2016). Rocking the boat ‐ Nursing students' stories of moral courage: A qualitative descriptive study. Nurse Education Today, 42, 35–40. 27237350 10.1016/j.nedt.2016.03.030

[nop21339-bib-0006] Bie, K. (2020). Refleksjon: Sykepleierens vei til klokskap [Reflection: Nurses' path to wisdom]. Universitetsforlaget.

[nop21339-bib-0007] Braun, V. , & Clarke, V. (2006). Using thematic analysis in psychology. Qualitative Research in Psychology, 3(2), 77–101.

[nop21339-bib-0008] Braun, V. , & Clarke, V. (2020). One size fits all? What counts as quality practice in (reflexive) thematic analysis? Qualitative Research in Psychology, 18, 328–352. 10.1080/14780887.2020.1769238

[nop21339-bib-0009] Cannaerts, N. , Gastmans, C. , & Dierckx de Casterlé, B. (2014). Contribution of ethics education to the ethical competence of nursing students: Educators' and students' perceptions. Nursing Ethics, 21(8), 861–878. 24714046 10.1177/0969733014523166

[nop21339-bib-0010] Dahl, H. , & Alvsvåg, H. (2013). Å fremme studenters evne til refleksjon–en pedagogisk utfordring [Promoting students' reflective ability: A pedagogical challenge]. Uniped, 36(3), 32–45.

[nop21339-bib-0011] Dahl, H. , & Eriksen, K. Å. (2016). Students' and teachers' experiences of participating in the reflection process “THiNK”. Nurse Education Today, 36, 401–406. 10.1016/j.nedt.2015.10.011 26556704

[nop21339-bib-0012] Dewey, J. (2008). Erfaring og opdragelse [Experience and upbringing] (2nd ed.). Reitzel.

[nop21339-bib-0013] Edwards, S. D. (2009). Nursing ethics: A principle‐based approach. Palgrave Macmillan.

[nop21339-bib-0014] Femdal, I. , Antonsen, E. B. , & Lepp, M. (2017). Studenters praksisrefleksjon med dramapedagogikk [Students' reflections on practice using drama teaching methodology]. Nordisk sygeplejeforskning, 7(2), 152–165. 10.18261/issn.1892-2686-2017-02-06ER

[nop21339-bib-0015] Gibson, E. , Duke, G. , & Alfred, D. (2020). Exploring the relationships among moral distress, moral courage, and moral resilience in undergraduate nursing students. Journal of Nursing Education, 59(7), 392–395. 10.3928/01484834-20200617-07 32598009

[nop21339-bib-0016] Hatlevik, I. K. R. (2018). Transformativ læring: Hva er det, og hva kan det bidra med i lærerstudenters kompetanseutvikling? [Transformative learning: What is it, and how can it improve student teachers' learning?]. Uniped (Lillehammer), 41(4), 384–400. 10.18261/issn.1893-8981-2018-04-02

[nop21339-bib-0017] Hazelwood, T. , Murray, C. M. , Baker, A. , & Stanley, M. (2017). Ethical tensions: A qualitative systematic review of new graduate perceptions. Nursing Ethics, 26(3), 884–902. 28905677 10.1177/0969733017727154

[nop21339-bib-0018] Heath, H. (1998). Reflection and patterns of knowing in nursing. Journal of Advanced Nursing, 27(5), 1054–1059. 9637334 10.1046/j.1365-2648.1998.00593.x

[nop21339-bib-0019] Heggestad, A. K. T. , Magelssen, M. , Pedersen, R. , & Gjerberg, E. (2020). Ethical challenges in home‐based care: A systematic literature review. Nursing Ethics, 28, 628–644. 10.1177/0969733020968859 33334250

[nop21339-bib-0020] Hunter, D. , McCallum, J. , & Howes, D. (2019). Defining exploratory‐descriptive qualitative (EDQ) research and considering its application to healthcare. GSTF Journal of Nursing and Health Care, 4(1). 10.5176/2345-7198_4.1.202

[nop21339-bib-0021] Karlsen, H. , Gjerberg, E. , Førde, R. , Magelssen, M. , Pedersen, R. , & Lillemoen, L. (2018). Etikkarbeid i kommunal helse‐ og omsorgstjeneste [Ethics work in primary healthcare]. Nordisk sygeplejeforskning, 8(1), 22–36. 10.18261/issn.1892-2686-2018-01-03ER

[nop21339-bib-0022] Karlsen, H. , Lillemoen, L. , Magelssen, M. , Førde, R. , Pedersen, R. , & Gjerberg, E. (2018). How to succeed with ethics reflection groups in community healthcare? Professionals' perceptions. Nursing Ethics, 26(4), 1243–1255. 10.1177/0969733017747957 29458313

[nop21339-bib-0023] Kim, W.‐J. , & Park, J.‐H. (2019). The effects of debate‐based ethics education on the moral sensitivity and judgment of nursing students: A quasi‐experimental study. Nurse Education Today, 83, 104200. 10.1016/j.nedt.2019.08.018 31491613

[nop21339-bib-0024] Koskinen, S. , Pajakoski, E. , Fuster, P. , Ingadottir, B. , Löyttyniemi, E. , Numminen, O. , Salminen, L. , Scott, P. A. , Stubner, J. , Truš, M. , Leino‐Kilpi, H. , & ProCompNurse Consortium . (2021). Analysis of graduating nursing students' moral courage in six European countries. Nursing Ethics, 28(4), 481–497. 10.1177/0969733020956374 33118442 PMC8182296

[nop21339-bib-0025] KS, the Norwegian Association of Local and Regional Authorities . (2021). Samarbeid om etisk kompetanseheving, verktøy og metoder [Collaboration to enhance knowledge of ethics: Tools and methods]. https://www.ks.no/fagomrader/helse‐og‐omsorg/eldreomsorg/samarbeid‐om‐etisk‐kompetanseheving/verktoy‐og‐metoder/

[nop21339-bib-0026] Løviknes, R. , & Struksnes, S. (2013). Indre motivasjon og ytre krav–Sykepleiestudenters utvikling av klinisk blikk [Internal motivation and external requirements: Nursing students' development of the clinical gaze]. Nordisk sygeplejeforskning, 3(3), 164–175.

[nop21339-bib-0027] Mæland, M. K. , Tingvatn, B. S. , Rykkje, L. , & Drageset, S. (2021). Nursing education: Students' narratives of moral distress in clinical practice. Nursing Reports, 11(2), 291–300. 10.3390/nursrep11020028 34968206 PMC8608063

[nop21339-bib-0028] Magelssen, M. , Gjerberg, E. , Pedersen, R. , Førde, R. , & Lillemoen, L. (2016). The Norwegian national project for ethics support in community health and care services. BMC Medical Ethics, 17(1), 70. 10.1186/s12910-016-0158-5 27825344 PMC5101716

[nop21339-bib-0029] Manninen, K. , Björling, G. , Kuznecova, J. , & Lakanmaa, R.‐L. (2020). Ethical coffee room: An international collaboration in learning ethics digitally. Nursing Ethics, 27(8), 1655–1668. 10.1177/0969733020934145 32666868

[nop21339-bib-0030] Martins, V. S. M. , Santos, C. M. N. C. , Bataglia, P. U. R. , & Duarte, I. M. R. F. (2021). The teaching of ethics and the moral competence of medical and nursing students. Health Care Analysis, 29(2), 113–126. 32944887 10.1007/s10728-020-00401-1 PMC8106588

[nop21339-bib-0031] Martinsen, K. (2021). Langsomme pulsslag [Slow heartbeats] (pp. 59–79). Fagbokforlaget.

[nop21339-bib-0032] Metselaar, S. , Molewijk, B. , & Widdershoven, G. (2015). Beyond recommendation and mediation: Moral case deliberation as moral learning in dialogue. The American Journal of Bioethics, 15(1), 50–51. 10.1080/15265161.2014.975381 25562230

[nop21339-bib-0033] Molewijk, A. C. , Abma, T. , Stolper, M. , & Widdershoven, G. (2008). Teaching ethics in the clinic: The theory and practice of moral case deliberation. Journal of Medical Ethics, 34, 120–124. 10.1136/jme.2006.018580 18234952

[nop21339-bib-0034] Nightingale, F. (2003). Håndbok i sykepleie: Hva det er og hva det ikke er [Notes on nursing: What it is and what it is not] (F. B. Larsen, Trans. Pensumtjeneste (Original work published 1859).

[nop21339-bib-0035] Nilsen, H. R. , Antonsen, Y. , Normann, A. K. , Kirkhaug, R. , & Tønnessen, S. (2015). Evaluering av det nasjonale prosjektet “Samarbeid om etisk kompetanseheving”. Styring, ledelse og gjennomføring . [Evaluation of the national project “Collaboration to enhance knowledge of ethics”. Management, leadership and implementation]. (Norut report 12/2015). https://norceresearch.brage.unit.no

[nop21339-bib-0036] Norwegian Ministry of Education and Research . (2017). Kultur for kvalitet i høyere utdanning [Culture for quality in higher education]. Lovdata. (White Paper No. 16 (2016–2017). https://www.regjeringen.no/no/dokumenter/meld.‐st.‐16‐20162017/id2536007/

[nop21339-bib-0037] Norwegian Ministry of Education and Research . (2019). Forskrift om nasjonal retningslinje for sykepleierutdanning [National nursing curriculum]. (FOR‐October 23, 2019‐1,405 fra 01.12.2019). Lovdata. https://lovdata.no/forskrift/2019‐03‐15‐412

[nop21339-bib-0038] Østerberg, D. (1966). Forståelsesformer: Et filosofisk bidrag [Forms of understanding: A philosophical contribution] (Vol. 314). Pax.

[nop21339-bib-0039] Pagnucci, N. , Carnevale, F. A. , Bagnasco, A. , Tolotti, A. , Cadorin, L. , & Sasso, L. (2015). A cross‐sectional study of pedagogical strategies in nursing education: Opportunities and constraints toward using effective pedagogy. BMC Medical Education, 15(1), 1–12. 26303930 10.1186/s12909-015-0411-5 PMC4549094

[nop21339-bib-0040] Paulsen, J. , Pedersen, R. , & Førde, R. (2011). Manual for kliniske etikk‐komiteer og etikkråd i kommunehelsetjenesten [Manual for clinical ethics committees and ethics councils in primary healthcare]. Centre for Medical Ethics, University of Oslo. https://www.med.uio.no/helsam/tjenester/kunnskap/etikk‐helsetjenesten/praksis/systematisk‐etikkarbeid/manual‐etikk‐kommunehelsetjenesten2011.pdf

[nop21339-bib-0041] Rasoal, D. , Skovdahl, K. , Gifford, M. , & Kihlgren, A. (2017). Clinical ethics support for healthcare personnel: An integrative literature review. HEC Forum, 29(4), 313–346. 10.1007/s10730-017-9325-4 28600658 PMC5688194

[nop21339-bib-0042] Sari, D. , Baysal, E. , Celik, G. G. , & Eser, I. (2018). Ethical decision making levels of nursing students. Pakistan Journal of Medical Sciences, 34(3), 724–729. 30034447 10.12669/pjms.343.14922 PMC6041523

[nop21339-bib-0043] Shorey, P. (1934). Aristotle, “The Metaphysics,” Books I‐IX. Hugh Tredennick. Classical Philology, 29(1), 84–85. 10.1086/361692

[nop21339-bib-0044] Shrivastava, S. , & Shrivastava, P. (2021). Encouraging reflection among medical undergraduate and postgraduate students for advancement of learning and development of skills. Journal of the Scientific Society, 48(2), 57–59.

[nop21339-bib-0045] Spekkink, A. , & Jacobs, G. (2021). The development of moral sensitivity of nursing students: A scoping review. Nursing Ethics, 28(5), 791–808. 10.1177/0969733020972450 33325340

[nop21339-bib-0046] Tutticci, N. , Coyer, F. , & Ryan, M. (2020). Optimising reflective capacity of nursing students after high fidelity simulation: A practical approach. Australian Journal of Clinical Education, 8(1), 1–17.

[nop21339-bib-0047] Vygotsky, L. S. (2001). Tenkning og tale [Thinking and speech] (T. Jarl‐Bielenberg, Trans. Gyldendal akademisk (Original work published 1934).

[nop21339-bib-0048] Wackerhausen, S. (2008). Erfaringsrum, handlingsbåren kundskab og refleksion [Experiential space, action‐based knowledge and reflection]. Department of Philosophy, Aarhus University.

[nop21339-bib-0049] Widdershoven, G. , Metselaar, S. , & Molewijk, B. (2016). Ethical theory as part of clinical ethics support practice. The American Journal of Bioethics, 16(9), 34–36. 10.1080/15265161.2016.1196260 27471936

